# Expanding Hereditary Spastic Paraplegias Limits: Biallelic *SPAST* Variants in Cerebral Palsy Mimics

**DOI:** 10.1002/acn3.70206

**Published:** 2025-09-26

**Authors:** Gregorio A. Nolasco, Mònica Roldán, Yalda Jamshidi, Ioannis Georvasilis, Rocío Jadraque Rodríguez, Reza Boostani, Ali Shoeibi, Lluís Armengol, Anna Codina, Ehsan Ghayoor Karimiani, Cristina Hernando‐Davalillo, Loreto Martorell, María Luisa Ramírez Almaraz, Jordi Muchart, Carlos Ortez, Andrés Nascimento, Roser Urreizti, Daniel Natera‐de Benito, Mercedes Serrano

**Affiliations:** ^1^ Pediatric Neurology Department Hospital Sant Joan de Déu Barcelona Spain; ^2^ Institut de Recerca Sant Joan de Déu Esplugues de Llobregat Barcelona Spain; ^3^ Confocal Microscopy and Cellular Imaging Unit, Genetic and Molecular Medicine Department Pediatric Institute for Rare Diseases Barcelona Spain; ^4^ Genetics Research Centre St George's, University of London London UK; ^5^ Genetics Centre of Excellence Novo Nordisk Research Centre Oxford Oxford UK; ^6^ Pediatric Neurology Department Hospital General Universitario Dr. Balmis Alicante Spain; ^7^ Department of Neurology Mashhad University of Medical Sciences Mashhad Iran; ^8^ Quantitative Genomic Medicine Laboratories, qGenomics Esplugues de Llobregat Barcelona Spain; ^9^ Applied Research in Neuromuscular Diseases, Department of Pathology Hospital Sant Joan de Déu Barcelona Spain; ^10^ Centre for Neuromuscular Diseases UCL Queen Square Institute of Neurology London UK; ^11^ Genetic and Molecular Medicine Department, Pediatric Institute for Rare Diseases Hospital Sant Joan de Déu Barcelona Spain; ^12^ U‐703 Center for Biomedical Research Network on Rare Diseases (CIBERER) Instituto de Salud Carlos III (ISCIII) Madrid Spain; ^13^ Diagnostic Imaging Department Hospital Sant Joan de Déu Barcelona Spain

**Keywords:** cerebral palsy, hereditary spastic paraplegias, pediatric neurology, *SPAST*, SPG4

## Abstract

**Objective:**

Hereditary spastic paraplegias (HSP) are rare neurodegenerative disorders marked by spasticity and lower limb weakness. The most common type, SPG4, is usually autosomal dominant and caused by *SPAST* gene variants, typically presenting as pure HSP. We describe five individuals from three unrelated families who meet the clinical criteria for cerebral palsy and carry biallelic *SPAST* variants. We aim to increase the clinical and genetic understanding of *SPAST*‐related disorders and explore the underlying abnormal cellular mechanisms.

**Methods:**

We performed comprehensive phenotyping and genetic analysis. *In silico* and functional studies were conducted using confocal microscopy on fibroblast cultures derived from carriers of the biallelic *SPAST* variants, a monoallelic *SPAST* variant, and a healthy control.

**Results:**

Individuals exhibited early‐onset complex HSP with a diverse range of encephalopathy severity, spasticity, and neuronoaxonal involvement, occasionally leading to the diagnosis of cerebral palsy. Whole‐exome sequencing identified homozygous and compound heterozygous *SPAST* variants. Functional studies demonstrated reduced spastin and tubulin levels, mitochondrial fragmentation, and abnormal filopodia morphology in patient‐derived fibroblasts, supporting the pathogenicity of the variants.

**Interpretation:**

We provide the first evidence of biallelic inheritance in *SPAST*‐related disorders, supported by functional analysis, expanding the clinical spectrum to include moderate‐to‐severe early‐onset encephalopathy. Our findings emphasize the importance of genetic diagnosis in cerebral palsy for prognosis, counseling, and personalized therapy. The identified variants reveal the genetic complexity of *SPAST*‐related disease and suggest a threshold effect of spastin levels in phenotypic variation. Cellular mechanisms such as mitochondrial dynamics and membrane morphology may contribute to pathogenesis and warrant further investigation.

## 1 Introduction

Hereditary spastic paraplegias (HSP) are a heterogeneous group of rare motor neurodegenerative disorders that are mainly characterized by a slow‐progressive bilateral spasticity and weakness of lower limbs [1, 2]. HSP, the second most common motor neuron disease, affects 3–10 out of every 100,000 people [3]. While spasticity is a constant feature in all cases of HSP, in certain types it is just one aspect of a wide range of symptoms, whereas in others, like SPG4, it is often the predominant clinical manifestation [4]. Over 100 loci/88 genes are known to be implicated in the pathogenesis of HSP [2, 3, 4, 5].

The most prevalent form of HSP is SPG4 (MIM #182601), caused by monoallelic variants in the *SPAST* gene (MIM *604277), which encodes spastin, a microtubule‐severing protein responsible for regulating different aspects of microtubule dynamics, such as their length, number, and mobility [6]. SPG4, also called *SPAST*‐related HSP, constitutes 40% of autosomal dominant HSP cases [7, 8]. SPG4 is distinguished as a ‘pure’ type of HSP, due to the predominance of spasticity without additional symptoms, although isolated reports speculate on other associated signs [9, 10, 11]. The *SPAST* gene contains 17 exons, within which 970 different mutations have been identified, scattered throughout the coding region [8]. While most of these are missense variants, deletions, duplications, and splicing variants (leading to loss of function alleles, LoF) also make a considerable contribution to the HSP pathogenesis. SPG4 patients bearing LoF variants are characterized by an indistinguishable clinical phenotype, suggesting that the molecular basis of the disease is haploinsufficiency. Conversely, recent evidence suggests an alternative possibility: The potential for dominant negative effects or a gain‐of‐function mechanism for some missense variants [12, 13]. Despite its prevalence, the molecular mechanisms underlying SPG4 are not fully understood. In fact, certain genotype–phenotype correlations have been suggested, including an earlier disease onset associated with missense variants located within the AAA domain [10]. To date, biallelic *SPAST* variants have been rarely reported, and have been associated not only with pure HSP [14, 15], but also with more severe phenotypes [15, 16, 17]. Their pathogenicity has been inferred based on clinical context and *in silico* predictions, yet remains unconfirmed, as no functional studies have been conducted to date in any of the reported cases [15, 16].

Complex and pure forms of HSP may be considered in the differential diagnosis of conditions referred to as cerebral palsy (CP) mimics or masquerades [18], in the absence of documented risk factors or neuroimaging findings consistent with a history of brain injury or a congenital cerebral malformation. Some genetic conditions that mimic CP may be considered developmental, while others are neurodegenerative. An accurate etiological diagnosis is essential, as identifying a genetic cause has significant implications for the patients and their families. Recent published studies indicate that between 15% and 30% of patients diagnosed with CP may have a genetic cause [19, 20]. This would increase the therapeutic options with a personalized approach [20], offer relevant information for the management, prognosis, and enable genetic counseling.

Here, we present five individuals from three unrelated families with biallelic *SPAST* variants, all exhibiting early onset complex HSP characterized by encephalopathy with diverse severity, spasticity, neuroaxonal involvement, and central nervous system neuroimaging abnormalities. We aim to support our clinical and molecular findings with functional studies, including evaluation of spastin and tubulin levels, mitochondrial fragmentation, and filopodia morphology. Furthermore, we seek to expand the current understanding of the inheritance patterns associated with *SPAST*‐related disorders. Additionally, our results broaden the molecular spectrum of *SPAST* variants, including a missense variant that profoundly alters the *SPAST* splicing pattern.

## 2 Methods

Five individuals (A1, B1, C1, C2, and C3) with biallelic *SPAST* variants underwent comprehensive phenotyping, including dysmorphology and general and neurological examinations (Table 1). The manuscript follows CARE guidelines.

**TABLE 1:** Clinical and molecular characteristics of the individuals.

Individuals	A1	B1	C1	C2	C3
Gender/origin	Female/Morocco	Male/Spain	Male/Iran	Male/Iran	Male/Iran
Variants (NM_014946)	c.1660A>G p.(Lys554Glu)/c.1660A>G p.(Lys554Glu)	c.1325A>T p.(Glu442Val)/c.1780C>T p.(Arg594Cys)	c.1370C>T p.(Ala457Val)/c.1370C>T p.(Ala457Val)		
Age at last examination	6 years	5 years 1 month	5 years 3 months	3 years 9 months	3 years 2 months
Occipito‐frontal circumference (percentile, SD)	48.5 cm (percentile < 1, −2.76 SD)	53 cm (percentile 90, 1.32 SD)	50 cm (percentile 10, −1.30 SD)	48.5 cm (percentile 4, −1.80 SD)	47.5 cm (percentile < 1, −2.38 SD)
Age at first symptoms	2 months	6 months	Neonatal	Neonatal	Neonatal
Maximum motor milestone acquired	No head lifting	Gait with frame	Rolling, no standing, no sitting	Rolling, no standing, no sitting	Rolling, no standing, no sitting
Language	Absent	Speech delay	Just babbling	Just babbling	Just babbling
ID/DD	Yes	Borderline	Yes	Yes	Yes
Spasticity	Yes	Yes	Clonus, Brisk 4+/Babinski+	Brisk 4+/Babinski+	Clonus, Brisk 4+/Babinski+
Spastic Paraplegia Rating Scale [21]	49	33	43	43	45
Dysphagia	Yes	No	Yes	Yes	Yes
MRI	Corpus callosum, thalamus and cerebellum atrophy. Volume loss of the posterior limb of the internal capsule	Normal	Mild white matter hyperT2 signal	NP	Normal
EMG/ENG	Axonal neuronopathy	Axonal neuronopathy	Axonal neuronopathy	NP	NP
VEEG^a^	Slow background activity No epileptic findings	Normal	Normal	NP	NP
Other findings			One large cafe‐au‐lait spot in back	One large cafe‐au‐lait spot, hyperstartle	Esotropia, camptodactyly
Carrier progenitors symptomatology	Father: mild spastic paraplegia Mother: no symptoms	No symptoms	Father: gait difficulties Mother: no symptoms		Not available

Abbreviations: EMG/ENG, Electromyogram/electroneurogram; ID/DD, Intellectual disability/developmental delay; MRI, Magnetic resonance imaging; SD, Standard deviation; VEEG, Videoelectroencephalogram.

^a^ No epileptic seizures in any of them.

### 2.1 Whole‐Exome Sequencing (WES), Variant Analysis, and *In Silico* Studies

DNA was isolated from venous blood samples of affected individuals and their parents using standard techniques. All patients were diagnosed by Next Generation Sequencing (NGS) at respective institutions, following established guidelines. Interinstitutional collaboration was facilitated via the GeneMatcher platform [22], The long form of the SPAST protein was used as a reference: NM_014946.4 (transcript) and Q9UBP0 (protein). To evaluate missense protein effects, several standard *in silico* tools were used (mainly, Polyphen2, SIFT, CADD, and DANN). PyMol was used for *in silico* visualization of the variants using the hexameric pdb model 6pen [23], and the monomeric AlfaFold model (AF‐Q9UBP0‐F1) [24, 25]. For conservation analysis, human SPAST was used as a BLAST query and model organisms were selected. Multiple protein alignment was performed using Clustal Omega [26].

### 2.2 Functional Studies

For the functional studies, five fibroblast culture lines were used: individual A1 is a 6‐year‐old female, homozygous for the p.(Lys554Glu) variant; subject B1 is a 5‐year‐old male, compound heterozygous for the p.(Glu442Val) and p.(Arg594Cys) variants; individual C1 is a 5‐year‐old male, homozygous for the p.(Ala457Val); subject “Het” is a 9 year old boy previously diagnosed with SPG4 and bearing the heterozygous pathogenic missense variant p.(Thr486Ile); and fibroblasts from a 3‐year‐old girl, as a healthy control.

Spastin was detected by indirect immunofluorescence using a monoclonal antibody (ab244354, Abcam, Waltham, MA, USA) and Fluor‐594 anti‐rabbit (A21207, Thermo Fisher Scientific Inc., Waltham, MA, USA). Alternatively, fibroblasts were stained with anti‐alpha tubulin (ab7750, Abcam) and Fluor‐488 anti‐mouse (A32766, Thermo Fisher). Nuclei were stained with Hoechst 33342 trihydrochloride trihydrate (Life Technologies, US) and mounted with Hydromount medium (National Diagnostics, UK).

Confocal microscopy analysis was performed with a Leica TCS SP8 equipped with a white light laser and Hybrid spectral detectors (Leica Microsystems, Mannheim, Germany), with images acquired using a HC × PL APO 63×/1.4 oil immersion objective. Hoechst 33342 was excited by a blue diode laser (405 nm) and detected within the 420–470 nm. Tubulin was excited using an argon laser (488 nm) and detected within the 505–550 nm. Spastin was excited with a white light laser (594 nm) and detected within the 615–795 nm. For spastin and tubulin quantification, Z stacks consisting of eight sections were acquired at 1 μm intervals throughout the cell thickness.

For live cell studies, fibroblasts were labeled with Hoechst 33342 (Life Technologies), and the plasma membrane was stained with CellMask Deep Red (Thermo Fisher) and Mitotracker Green FM (Life Technologies). Hoechst 33342 DNA label was excited by a blue diode (405 nm) and detected within the 415–460 nm range. MitoTracker Green FM was excited using an Ar laser (488 nm) and detected in the 520–555 nm range. CellMask was excited with a white laser (633 nm) and detected within the 650–795 nm range. High‐speed 3D time‐lapses were recorded using the same microscope in the high‐speed acquisition mode with a 63× (NA 1.4 oil) Plan‐Apochromatic objective. Projections were generated from 15 serial optical sections (z‐step = 0.7 μm) acquired every 22 s over a period of 10 min. Image deconvolution was performed using Huygens Professional software v17.10.0p7 64b (SVI, Leiden, The Netherlands).

Mitotracker Green staining was used to assess mitochondrial morphology and distribution patterns in cells from affected individuals. The *Mitochondria Analyzer* plugin in Fiji/Image J was used to analyze mitochondria from patient‐derived fibroblasts [27]. Video data were loaded, and the first frame corresponding to the initial time point was selected. The image was converted to 8‐bit, and the scale was set to microns (1 px = 0.09 μm) for accurate measurements. Three representative images were chosen for threshold optimization, adjusting the C‐value (7) and block size (1.45) to achieve the best binarization. The thresholding was applied using rolling subtraction (1.25), sigma radius (1.11), enhancement max (1.80), gamma adjustment (0.80), and a weighted mean method. After binarization, a 2D morphological analysis of the mitochondria was performed using the *Mitochondria Analyzer* plugin, quantifying parameters such as count, area, perimeter, shape, aspect ratio, and various branching parameters. Mean values (total mean, mean_0, mean_1) and branching parameters (branch_0, branch_1, branch_2) were also assessed. These measurements provided a comprehensive evaluation of mitochondrial morphology and cellular integrity, essential for understanding the impact of biallelic *SPAST* variants.

### 2.3 Statistical Analysis

The data are shown as means ± SEM and visually represented through either column bars, complemented with error bars. Significance levels are denoted by asterisks: *p < 0.05, **p < 0.01, ***p < 0.001, and ****p < 0.0001. Graphs and statistical analysis were performed using GraphPad Prism version 8.0.1 (GraphPad Software Inc., La Jolla, CA). To assess the normality of the data, the Shapiro–Wilk test was performed. To analyze overall differences among all samples, the nonparametric Kruskal–Wallis test was used. Pairwise comparisons, both for each patient and against control data, were conducted using the Mann–Whitney test (for non‐normally distributed data).

### 2.4 Ethics Approval and Consent to Participate

Signed Informed Consent was obtained from the parents/caregivers of study participants. Data were collected in accordance with ethical guidelines established by the institutions involved. Ethical standards laid down were in accordance with the Helsinki Declaration of 1964, as revised in October 2013 in Fortaleza, Brazil. The protocol was approved by the Research and Ethics Committee of the SJD Research Foundation (protocol ID PIC 41‐18).

## 3 Results

### 3.1 Clinical Descriptions

**FIGURE 1:**
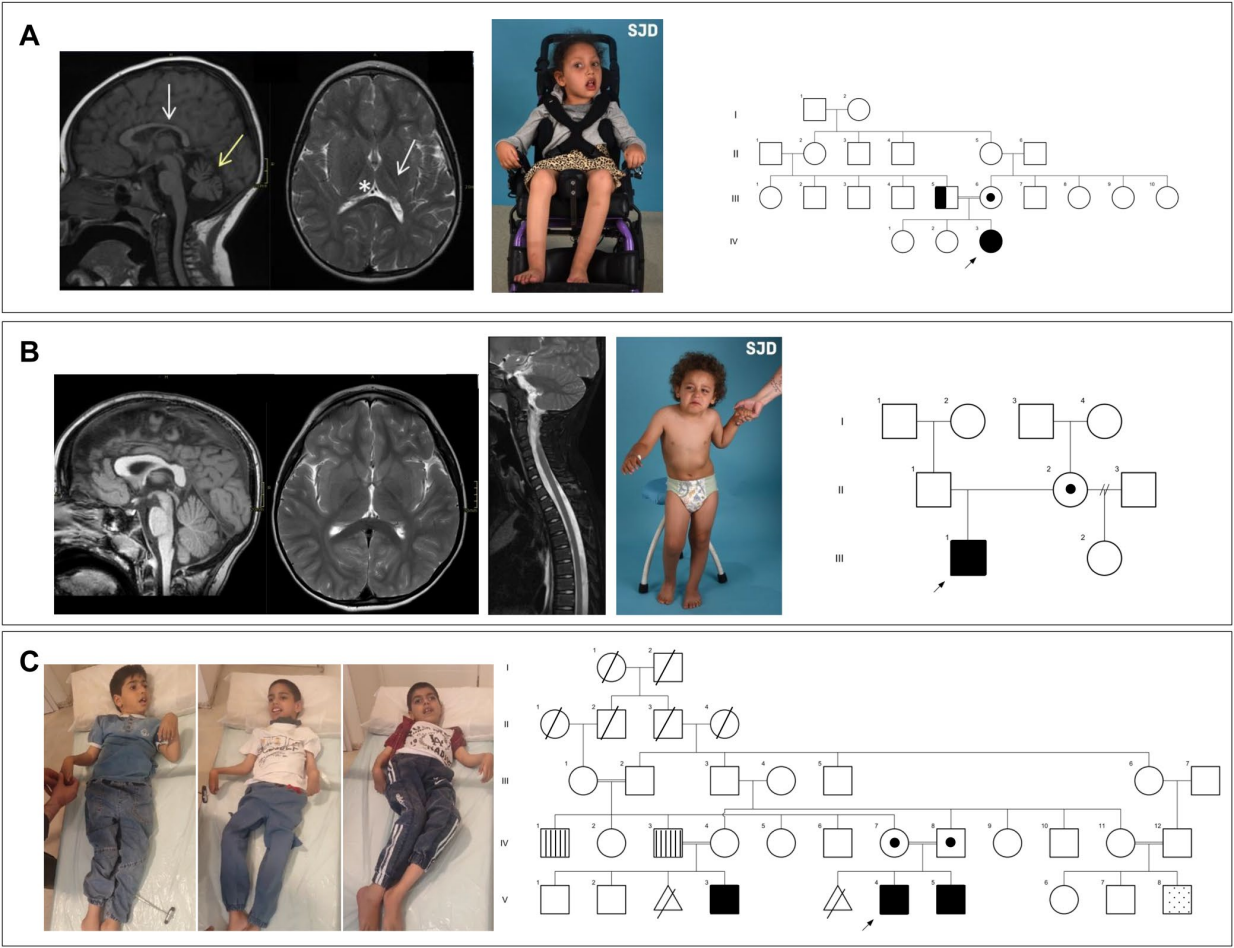
Pedigree electropherograms, clinical features and neuroimaging. (A) From left to right: Brain MRI of individual A1 at 5 years of age. Sagittal FLAIR T1 (left) and axial FSE T2 (right). Corpus callosum thinning (white arrow) and cerebellar atrophy (yellow arrow) can be observed. Note also severe volume loss and high T2 intensity of the posterior limb of the internal capsule (arrow) and thalamic atrophy (asterisk). Whole body and facial pictures of individual A1 showing severe motor impairment, hypomimics with oral hypotonia. Right, the pedigree shows consanguinity between individuals III 5 and III 6, both heterozygous carriers of the variants and only III 5 being symptomatic. (B) From left to right: MRI of individual B1 at 4 years of age. Brain sagittal 3D TFE T1 (left) and axial FSE (middle). Normal corticospinal tract volume and signal and appropriate thalamic volume. No corpus callosum or cerebellar atrophy are found. Spinal sagittal FSE T2 (right) showing spinal cord volume within normal values, without signal abnormalities. Whole body pictures of individual B1 showing abnormal standing position requiring support. The pedigree shows case II 2 was a carrier of the variant, with a black arrow identifying the proband (III 1). (C) From right to left: Whole body pictures of individuals C1, C2, and C3. Extensive history of consanguinity was identified in family 3. Cases IV 1 and IV 3 presented poor hearing. Individual V 8 case presented gait difficulties. The probands V3 (individual C3), V4 (individual C1), and V5 (individual C2) presented progressive spasticity.

We describe five individuals from three families, bearing homozygous or compound heterozygous variants in *SPAST*. All presented with peripheral and central symptoms, gross and fine motor delay, communication delay, and cognitive impairment during the first 6 months of life leading to an initial diagnosis of CP. Table 1 and Figure 1 encompass the clinical and molecular data of the individuals.

#### 3.1.1 Family A

The proband (individual A1) is a 6‐year‐old Moroccan female born to consanguineous parents. Family history was unremarkable for neurological disorders. She was born following a third uneventful pregnancy and delivery. From early infancy, she exhibited axial hypotonia and global developmental delay, with progressive spasticity presenting around 12 months of age. She has never acquired adequate head support, independent sitting, or the ability to stand upright. At 5 years 10 months, she underwent Nissen fundoplication, and feeding is exclusively performed via gastrostomy due to severe dysphagia. She exhibits speechlessness and a gradual decline in cognitive function and response speed. She has never presented epilepsy.

Regular examinations between 4 and 6 years old revealed marked axial hypotonia associated with spastic tetraparesis. Generalized hyperreflexia, bilateral extensor plantar response, and sustained ankle clonus were also observed. Eye contact was poor, with difficulty following faces and objects and a tendency to upward gaze. No dysmorphic features were noted, although marked hypomimia was observed. The Spastic Paraplegia Rating Scale assessment yielded a score of 49 points [21].

MRI scans performed at 2 and 5 years of age revealed a thin corpus callosum, loss of volume of both sides of the thalamus, gliosis of the internal capsule, and a marked atrophy of the cerebellum (Figure 1A). Motor and sensory nerve conduction studies were normal. EMG shows a neurogenic pattern. Auditory evoked potentials were normal, and visual evoked potentials showed a transmission delay. The electroencephalogram (VEEG) performed at 5 years 7 months showed slow background brain activity without epileptiform activity. Standard cognitive evaluation was impossible due to severe neurological involvement. After learning the diagnosis at the physical exam, the father showed mild hypertonia in the lower extremities, with patellar hyperreflexia, an increase in the reflexogenic area, and mild Achilles retraction. His motor and sensory nerve conduction studies were normal, and his EMG showed a neurogenic pattern.

#### 3.1.2 Family B

Individual B1 is a 5‐year‐old Spanish male born to healthy, non‐consanguineous parents following an uncomplicated pregnancy and delivery. His 12‐year‐old half sister from the mother's side is healthy and does not show spasticity or hyperreflexia. Psychomotor delay was first noted when he was 6 months old, and progressive lower limb spasticity was first observed between 1 and 2 years of age. At 4 years old, he had acquired stable sitting but had not achieved independent ambulation.

The physical examination conducted at 5 years of age showed spastic paraparesis and axial hypotonia. He has a generalized hyperreflexia and bilateral equivocal plantar responses, with no evidence of clonus. No dysmorphic features are observed. He is able to eat solid food and only rarely experiences choking episodes. He has not achieved sphincter control. He attends mainstream school with educational adaptations. He currently has significant language difficulties, starting to construct short sentences at the age of four. He understands simple questions and identifies body parts, and recognizes some colors. Cognitive evaluation using WPPSI‐IV (Wechsler Preschool & Primary Scale of Intelligence IV) and ABAS II (Adaptive Behavior Assessment System‐II) at 5 years and 9 months of age showed a total intelligence quotient of 83 (verbal comprehension: 77, fluid reasoning: 103, processing speed: 85, visual spatial reasoning: 82, and working memory: 81), with a notable impairment in expressive communication skills. In this patient, the Spastic Paraplegia Rating Scale assessment yielded a score of 33 points [21].

Brain and spinal MRI at 3 years old was normal (Figure 1B). Motor and sensory nerve conduction studies were normal. EMG shows a neurogenic pattern.

#### 3.1.3 Family C

Individual C1 is a 5‐year 3‐month‐old male, born following a second uncomplicated pregnancy and delivery. The parents, of Arab ethnic origin, were healthy and consanguineous, with a history of a previous abortion. No resuscitation was required at birth. However, hypotonia was observed in the immediate perinatal period. At the age of 5 years, he exhibits hypertonia, spasticity, and bilateral Babinski reflex. His highest motor milestone achieved is rolling, and his speech is limited to cooing. Brain MRI at 4 years showed mild white matter hyperintensity T2 signals (nonspecific), and video‐EEG was normal.

Individual C1 has a younger brother, individual C2, a 3‐year 9‐month‐old male, who also presents with severe developmental delay and spasticity, similar to C1, with hyperstartle responses. Additionally, a cousin affected by developmental delay (individual C3) was also identified, showing global motor and cognitive delay, microcephaly, strabismus, and camptodactyly. MRI of individuals C2 and C3 yielded normal results. The three individuals exhibit a severe neurological syndrome characterized by hyperreflexia, generalized spasticity, and hypomimia (Figure 1C). None of them have achieved independent gait, and they present with mild‐to‐moderate intellectual disability in the absence of epilepsy. These patients scored 43, 43, and 45, respectively, on the Spastic Paraplegia Rating Scale [21].

### 3.2 Molecular Results and *In Silico* Evaluations

Blood analysis, including extensive metabolic workup and CGH‐array analysis, was performed, with normal results for all individuals.

**FIGURE 2:**
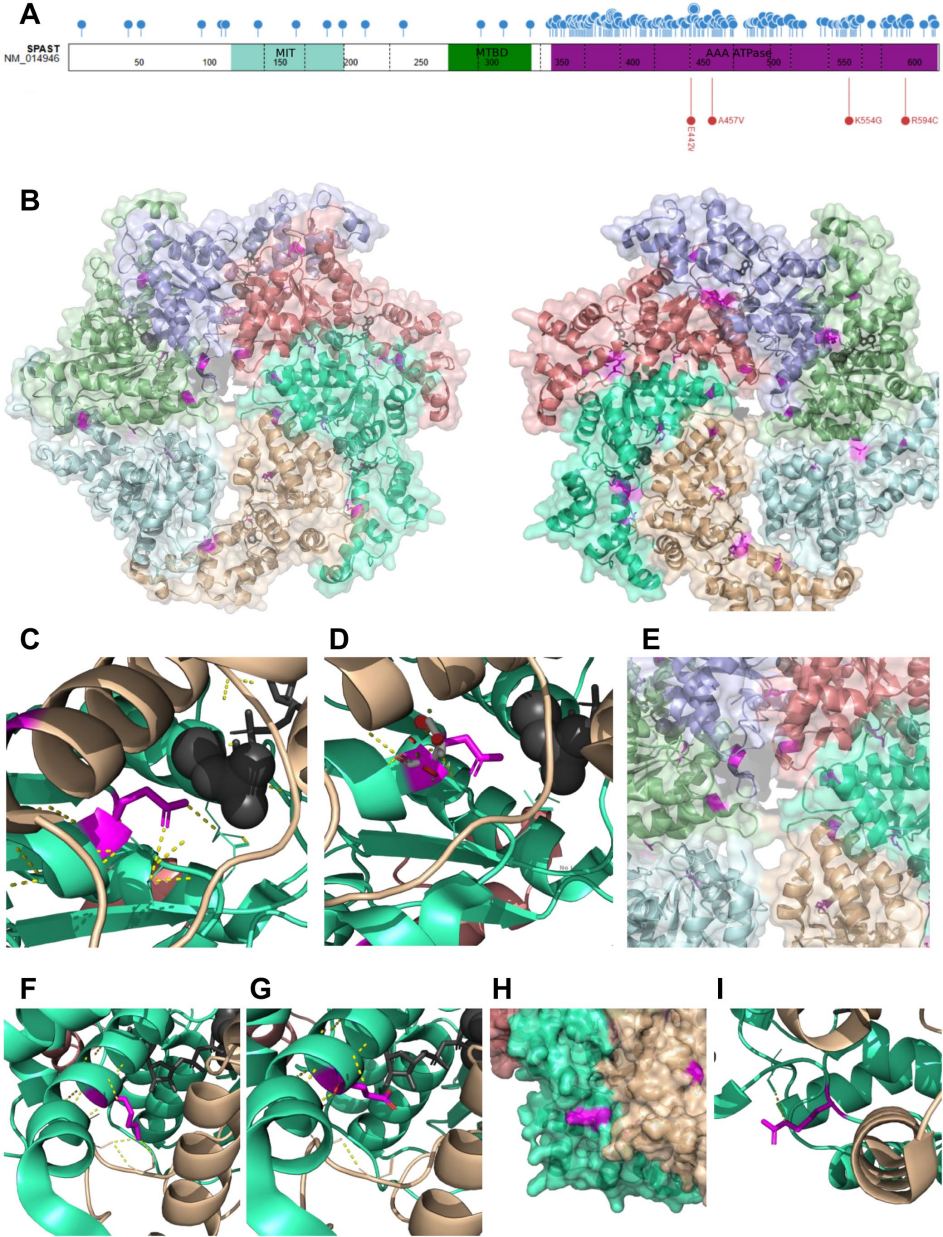
*In silico* analysis of spastin mutations and structure. (A) Schematic representation of spastin monomer indicating the previously described missense variants collected in HGMD Pro (up, blue) and the variants identified here (down, red). Dotted lines indicate exon boundaries. MIT, MTBD and AAA ATPase domains are represented in turquoise, green and magenta respectively. (B) Visualization of the spastin hexamer (each monomer a different color, pdf model 6pen) and ligands (ADP and ATP, in gray), top and bottom views with the variants identified here in magenta. (C) Close‐up of Glu442 and polar interactions with Asn487 and Thr486 of the same monomer in close vicinity to ATP (gray). (D) Substitution of Glu442 by Val (in white) present steric clashes (indicated as red discs) suggesting an effect on the 3D structure of the protein. (E) Close‐up of Ala457 residue, in close vicinity to the spastin complex central pore. (F, G) Close up of Lys554 showing polar interaction with Ala373 of the adjacent spastin subunit in close vicinity to the ADP molecule (in gray) (D) and model substitution by Glu (E). (H) Close‐up and surface representation of the Arg594 region. (I) Arg594 is predicted to be involved in polar contacts with Val596 of the same monomer.

WES analysis revealed the homozygous *SPAST* variant c.1660A>G; p.(Lys554Glu) in individual A1, compound heterozygous *SPAST* variants c.1325A>T; p.(Glu442Val) and c.1780C>T; p.(Arg594Cys) in individual B1, and homozygous *SPAST* variant c.1370C>T; p.(Ala457Val) in individual C1 (later confirmed in C2 and C3). All variants are absent from reference population databases (gnomAD 4.0 accessed April 2025, see Table S1), affect highly conserved residues, and were predicted as damaging by *in silico* tools (see Figure S1). Variant p.(Ala457Val) has been previously reported in the literature [28], while p.(Glu442Val) affects a residue in which other SPG4 pathogenic variants have been identified [29, 30, 31]. All the variants described here were classified as Likely Pathogenic according to the ACMG criteria [32]. They are all located near previously reported variants, all within the AAA cassette (Figure 2A). The position of the four variants identified here on the Spastin hexamer has been assessed on the hexamer model of spastin complex [23] (Figure 2B). Amino acid Glu442 has been determined to be part of the Walker B domain and it has been previously determined that it is essential for spastin function [33, 34]. This residue lays in close vicinity to ATP (Figure 2C) and is predicted to be involved in polar contacts with neighboring residues Thr486 and Asn487 of the same monomer. The substitution of the acidic and negatively charged Glu by the non‐charged Val is predicted to have a major effect on protein function and structure (Figure 2D). Variant p.(Ala457Val) (Figure 2C) substitutes an Ala residue laying on an alpha‐helix in close vicinity to the microtubule aperture by Val, a C_β_ branched amino acid which could destabilize alpha‐helix conformation [35]. Lys554, in close vicinity to the ADP molecule of the monomer, is also predicted to be involved in polar interactions with Ala373 of the adjacent monomer (Figure 2F) and its substitution by Glu (as in individual A1) would disturb those interactions (Figure 2G). Finally, p.(Arg594Cys) involves the substitution of the basic and positively charged Arg in the external surface of the structure (Figure 2H) by the hydrophilic and non‐charged Cys and this substitution is predicted to abolish Arg594 polar interaction with Val596 of the same monomer (Figure 2I).

No additional variants of interest in relevant disease‐causing genes were found in any of the three individuals. Segregation analysis was performed by Sanger sequencing. In families A and C, parents were carriers of a monoallelic *SPAST* variant. In family B, the mother carried the variant c.1780C>T while the father did not bear the variant c.1325A>T (Figure 1). The paternal relationship was confirmed by microsatellite analysis, indicating that this second change occurred *de novo*. To confirm that the *de novo* variant lies on the paternal chromosome and results in compound heterozygosity, both variants were analyzed at the mRNA level in fibroblasts from individual B1. Exons 11–17 of the SPAST transcript, encompassing both variants, were amplified from the patient's cDNA and sequenced. The maternal allele (c.1780C>T in exon 17) was detected in hemizygous state (appearing as homozygous), while the paternal variant (c.1325A>T in exon 11) was not detected at the mRNA level (Figure S2). This finding supports the presence of compound heterozygosity and suggests that the paternal c.1325A>T variant leads to a splicing defect, as predicted by HSF algorithms [32].

### 3.3 Functional Studies

#### 3.3.1 Spastin and Tubulin Immunofluorescence

**FIGURE 3:**
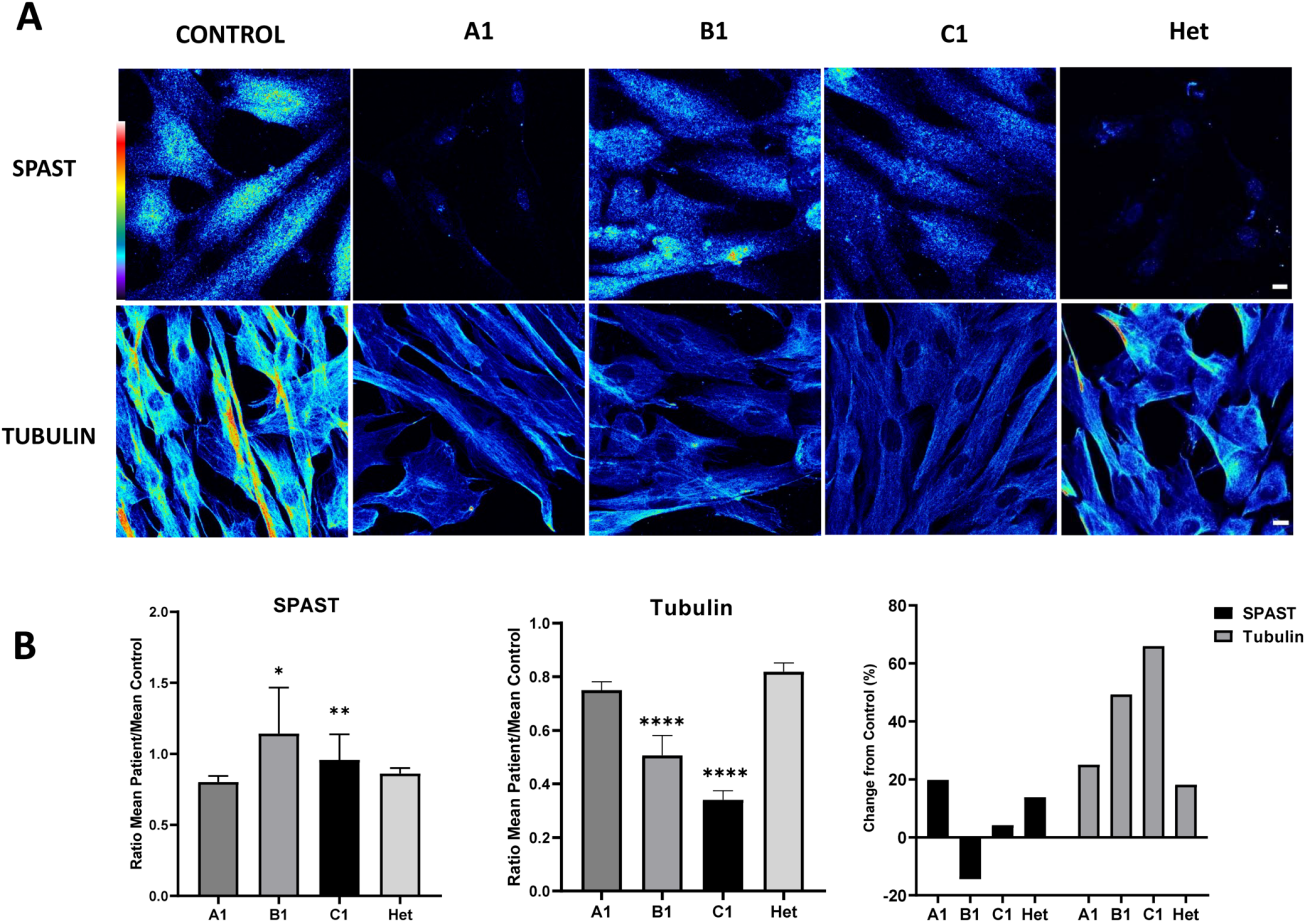
Spastin and α‐tubulin expression in fibroblasts. (A) Confocal microscopy images of fibroblasts illustrate the expression of spastin and α‐tubulin. A pseudocolor palette was used to visualize different intensity levels in the spastin and α‐tubulin signals. Warm colors (white to red scale) indicate higher intensities, while cool colors (blue) represent lower intensities. The pseudocolor scale facilitates comparison, revealing reduced fluorescence intensity in the patient samples compared to the control. This scale is shown in the top left corner of the figure. Scale bar: 10 μm. (B) Graphical representation of the mean fluorescence intensity ratios (patient vs. control) for spastin and α‐tubulin. In both cases, patients generally show reduced mean intensity ratios compared to healthy controls. Data are presented as mean ± SEM. Statistical significance is indicated as follows: *p < 0.05, **p < 0.01, and ****p < 0.0001. As additional information, the standard deviation (SD) of the individual intensity ratios was as follows: For spastin, A1 = 0.0955, B1 = 1.0204, C1 = 0.7010, Het = 0.0872; and for α‐tubulin, A1 = 0.1095, B1 = 0.2345, C1 = 0.1328, Het = 0.1146. Percentage changes in SPAST and α‐tubulin levels relative to controls were calculated as: [100 − (Patient mean × 100/Control mean)], indicating the relative decrease in protein levels compared to controls.

Overall, the immunofluorescence analysis revealed a decrease in the signal intensity of spastin (SPAST) and α‐tubulin in patients' fibroblasts compared to control cells. Specifically, fibroblasts from individual A1 (homozygous for the p.(Lys554Glu) variant) exhibited a 19.8% reduction in SPAST intensity, while in the Het individual, who carries the monoallelic p.(Thr486Ile) variant associated with SPG4, the decrease was 13.8% compared to controls (Figure 3B). In fibroblasts from individual A1 and Het, spastin localization was predominantly nuclear, displaying a punctate pattern. On the other hand, patients B1 and C1 showed a spastin fluorescence intensity very similar to controls, although a slight reduction of 4.2% was detected in C1 (Figure 3B). In these two patients, spastin was distributed throughout the entire cell, including both the nucleus and cytoplasm.

Regarding α‐tubulin, fibroblasts from individual A1 showed a 25.0% reduction, those from patient B1 displayed a more pronounced reduction of 49.3%, and fibroblasts from patient C1 showed the greatest decrease, with 65.97% (Figure 3B). Fibroblasts from the Het individual exhibited an 18.2% decrease compared to controls (Figure 3B).

#### 3.3.2 Mitochondrial and Filopodia Morphology

**FIGURE 4:**
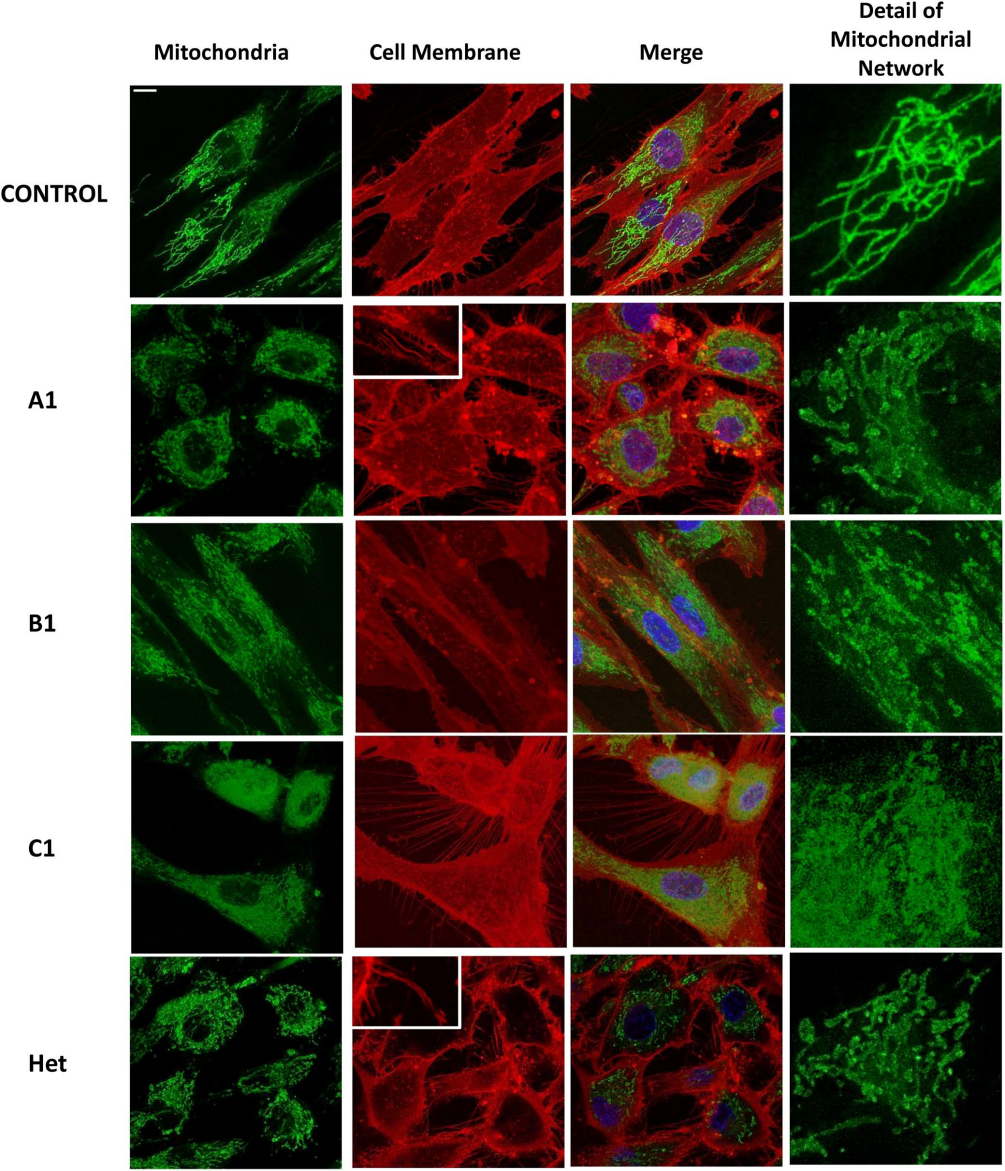
Mitochondrial and cell membrane morphology. Mitochondrial and cell membrane morphology. Representative confocal images of fibroblasts derived from control and patients, labeled with MitoTracker Green (mitochondria, green), CellMask Deep Red (cell membrane, red), and Hoechst (nuclei, blue). Scale bar: 10 μm. The inset shows the differences in filopodia‐like structures morphology between heathy controls and patients. Detail of the mitochondrial network is shown on the right.

**FIGURE 5:**
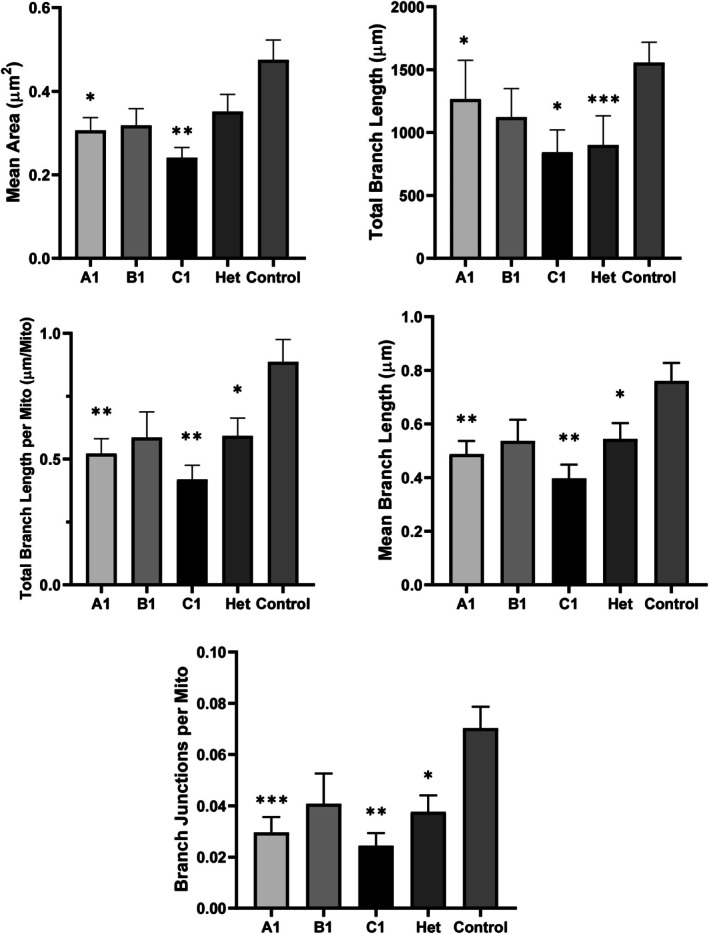
Boxplots of mitochondrial morphological parameters in patients and controls. Mean area represents the average mitochondrial area per cell; Total branch length refers to the total length of the mitochondrial network; Total branch length per mitochondrion reflects the average extension of individual mitochondria; Mean branch length indicates the average length of each branch; and Branch junctions per mitochondrion measures mitochondrial connectivity, with lower values indicating reduced interconnection. Data are presented as mean ± SEM. Statistical significance is indicated as follows: *p < 0.05, **p < 0.01, and ***p < 0.001. As additional information, the standard deviation (SD) for individual patients was: For Mean area: A1 = 0.1156, B1 = 0.0900, C1 = 0.0684, Het = 0.1605; for Total branch length: A1 = 1159.4231, B1 = 506.8811, C1 = 503.8802, Het = 896.2220; for Total branch length per mitochondrion: A1 = 0.2243, B1 = 0.2276, C1 = 0.1601, Het = 0.2776; for Mean branch length: A1 = 0.1844, B1 = 0.1758, C1 = 0.1469, Het = 0.2278; and for Branch junctions per mitochondrion: A1 = 0.0224, B1 = 0.0264, C1 = 0.0140, Het = 0.0247.

Patient fibroblasts exhibited a smaller average mitochondrial area and shorter branch length as noted by the Mitochondria Analyzer plugin, along with a reduction in all the studied morphological parameters compared to the control. The staining revealed highly fragmented and small mitochondrial structures, with low interconnectivity and branching, features especially evident in the cells of individuals A1 and C1 (Figures 4 and 5), indicating a dysfunctional mitochondrial network.

Specifically, the mean mitochondrial area was reduced in all patient‐derived fibroblasts compared to controls, with the most pronounced decreases observed in individuals A1 and C1. Statistically significant reductions in mitochondrial area were observed in A1 and C1, whereas the decrease observed in Het did not reach statistical significance. Regarding Total Branch Length, which represents the total space occupied by the mitochondrial network in terms of length, the most substantial reductions were observed in Het and C1, with significant decreases found in all three patient‐derived fibroblast lines (A1, C1, and Het) compared to the control.

In terms of Total Branch Length per Mitochondrion, individuals A1 and C1 exhibited the shortest extensions, both of which were statistically significant. Similarly, A1 and C1 demonstrated the lowest values in Mean Branch Length (average size of a mitochondrial branch) as well as Branch Junctions per Mitochondrion, both parameters showing statistically significant reductions. These findings reflect a markedly reduced level of mitochondrial interconnectivity. Although less pronounced than in A1 and C1, Het also exhibited statistically significant decreases in these metrics compared to controls.

In contrast, fibroblasts from healthy control exhibited longer and more highly interconnected mitochondria, consistent with a well‐organized and functional mitochondrial network. Statistically significant differences were observed between control cells and patient‐derived lines (A1, C1, and Het) across all parameters, with the exception of mean mitochondrial area in Het, which did not reach statistical significance (Figure 5). Additionally, a higher concentration of mitochondria around the perinuclear region was observed in fibroblasts derived from affected individuals (Figure 4). In terms of membrane morphology, both the cells of individual A1 and individual Het showed a significant number of long and thick filopodia‐like structures that failed to adhere to the substrate (Figure 4, Videos S1–S3), suggesting impaired adhesion capacity.

Taken together, these findings highlight distinct and statistically significant differences in mitochondrial and filopodia‐like structures' morphology between patient‐derived and control fibroblasts.

## 4 Discussion

This study provides novel insights into the clinical manifestations, molecular, and cellular mechanisms associated with biallelic variants in the *SPAST* gene among individuals presenting with moderate‐to‐severe early‐onset encephalopathy, spastic tetraparesis, and neuronoaxonal involvement. Our findings broaden the understanding of *SPAST*‐related disorders, which have been predominantly characterized by monoallelic mutations leading to the autosomal dominant SPG4 form.

The identification of biallelic *SPAST* variants and individuals exhibiting CP mimics challenges existing paradigms and suggests a recessive inheritance pattern, expanding the pool of genes implicated in CP or complex HSP. Recent studies support the routine use of chromosomal array and exome sequencing in all patients presenting with cerebral palsy [20]. These methods show a diagnostic yield comparable to that seen in other neurodevelopmental disorders, even within diverse and unselected patient cohorts [36]. While genetic diagnosis may not offer immediate targeted therapies, it significantly enhances patient care by providing essential prognostic insights and facilitates genetic counseling, especially for individuals with HSP mimicking CP.

Cruz‐Camino et al. [15], previously reported early onset and rapid progression associated with biallelic variants in *SPAST* in two siblings carrying a homozygous variant p.(Ser545Ter). These siblings displayed a severe phenotype characterized by spastic paraplegia, rapid psychomotor deterioration, and cerebellar hypoplasia. More recently, Degoutin et al. [16], described a series of nine patients carrying biallelic *SPAST* variants with variable age of onset and clinical expression ranging from psychomotor regression to childhood neurodegenerative disorder associated with tetraparetic syndrome. In this latter study, MRI findings were unremarkable or unspecific, both in supratentorial and infratentorial structures.

Our cohort analysis consistently demonstrates the involvement of encephalic structures of varying severity among the five individuals with biallelic *SPAST* variants. This is characterized by global developmental delay from infancy, progressive spasticity, and varying degrees of cognitive impairment. Neuroimaging revealed nonspecific findings in individual C1, while striking microcephaly and cerebellar atrophy were observed in individual A1, the most severely affected individual in the cohort. Notably, all individuals exhibited some degree of intellectual disability. These findings suggest that a range of neurodevelopmental factors beyond gross anatomical abnormalities may contribute to this novel SPAST‐related phenotype, potentially including subtle microstructural alterations, synaptic dysfunction, or neurotransmitter imbalances. Remarkably, our findings highlight a progressive encephalopathy affecting both infratentorial and supratentorial structures. The standardized evaluation of our patients using the Spastic Paraplegia Rating Scale yielded scores ranging from 33 to 49 points. Unfortunately, this valuable metric is not available for comparison in previous reports, as such data were not provided. Moreover, in all the aforementioned reports describing more severe phenotypes linked to SPAST, functional studies supporting the pathogenicity of the homozygous *SPAST* variant are unfortunately absent, preventing the definitive establishment of the variant as the causal factor of their severe neurodegenerative conditions. For the first time, our study provides strong evidence of functional abnormalities.

Dominant HSP subtypes are more prevalent than recessive ones. While the list of genes contributing to recessive HSP is expanding, some subtypes remain constrained to specific families or individuals [37]. Certain genes, including *ATL1*, *ALDH18A1*, and *KIF1A*, exhibit both dominant and recessive inheritance patterns linked to HSP [38, 39, 40]. Incorporating the *SPAST* gene into this framework enriches our understanding of dual inheritance patterns within HSP. A plausible hypothesis to explain the dominant and recessive inheritance linked to *SPAST* is that the variants in the individuals described here function as hypomorphs, generating reduced levels of product activity. When in heterozygosity, the amount of spastin is not sufficiently altered to give rise to symptoms. Consequently, a “threshold effect” of spastin levels could be at play, thereby also accounting for the diversity in severity among individuals with monoallelic variants. The variant reported here, p.(Ala457Val), has been previously described in the literature [28]. Variant c.1325A>T has been shown to alter the splicing pattern and lead to a null allele, but if translated, p.(Glu442Val) affects a residue in which a pathogenic variant has previously been identified in individuals with pure SPG4 [29]. The presence of a *de novo* potentially LoF variant in compound heterozygosity highlights the need to further unravel the genetic landscape related to *SPAST*.

Biallelic *SPAST* variants do not follow a specific distribution pattern and are scattered across the AAA domain, similarly to monoallelic variants [14, 15, 16, 17]. Truncating and missense variants have both been linked to SPG4. The absence of biallelic combinations of variants causing SPG4 with dominant inheritance suggests potential lethality. Conversely, variants causing mild symptoms in a heterozygous state might lead to severe phenotypes when biallelic. This may be the case for the carrier parents of our cohort. However, additional experimental exploration is needed to confirm this emerging hypothesis.

Our functional investigations in fibroblast cultures elucidated the effects of genetic variants on protein distribution and cellular morphology. Confocal studies revealed a loss of microtubules (MTs), which play a crucial role in cellular organization, intracellular trafficking, and organelle positioning. Spastin, an enzyme responsible for cleaving long MTs into shorter fragments, contributes to MT stability and function [6, 31]. Variants in the *SPAST* gene appear to impair spastin activity, leading to disruptions in the microtubule network. Our findings show that patients with homozygous mutations (A1 and C1) exhibit lower spastin and tubulin levels, resulting in significant microtubule destabilization. In contrast, heterozygous patients (Het) show milder effects, indicating a potential threshold effect in spastin activity reduction. The differences in microtubule stability between patient groups suggest that specific mutations may differentially affect spastin localization and function, leading to variable cellular consequences.

The disruption of microtubules in patient fibroblasts correlates with mitochondrial abnormalities. This may be explained by the fact that MTs are essential for proper mitochondrial distribution, morphology, and intracellular trafficking [6, 41, 42, 43]. Our findings indicate a fragmented mitochondrial network, particularly in fibroblasts from homozygous patients, where low interconnectivity and branching were observed compared to healthy controls. Additionally, a higher concentration of perinuclear mitochondria in patient‐derived fibroblasts suggests alterations in intracellular trafficking. The most severe mitochondrial defects were observed in patients A1 and C1, likely due to impaired mitochondrial transport caused by disrupted microtubule dynamics. These findings support the hypothesis that mitochondrial dysfunction in these patients is directly linked to microtubule instability and spastin impairment. Recent research has uncovered novel molecular mechanisms of spastin in various cellular pathways, including endoplasmic reticulum conformation, calcium and fatty acid trafficking, fission, and endosomal trafficking [6, 41, 42].

Filopodia‐like structures, actin‐driven projections essential for cell movement and adhesion, also exhibit significant morphological alterations in patient fibroblasts [43]. The coordination between actin filaments and MTs is crucial for filopodia formation and function [44, 45]. In our study, cells from patients A1 and Het displayed an increased number of long, thick filopodia‐like structures that failed to adhere to the substrate (Figure 4, Videos S1–S3). This may be due to a decoupling of the interaction between MTs and filopodia, disrupting cytoskeletal remodeling [46]. Additionally, nuclear mislocalization of spastin in these patients may further interfere with actin cytoskeleton regulation, leading to impaired cellular movement and adhesion. These results highlight the broader consequences of spastin dysfunction beyond microtubule destabilization, affecting multiple cytoskeletal elements.

Genotypic analysis further supports the link between *SPAST* variants and the observed cellular abnormalities. Patient A1 carries the homozygous c.1660A>G; p.(Lys554Glu) mutation, which may lead to a loss of cytoplasmic localization, causing nuclear accumulation and impairing spastin's normal role in microtubule regulation [41]. Patient C1, with the homozygous c.1370C>T; p.(Ala457Val) mutation, also exhibits severe mitochondrial defects, suggesting a strong impact on microtubule stability and intracellular transport [41]. In contrast, patient B1, who carries two different heterozygous mutations, c.1325A>T; p.(Glu442Val) and c.1780C>T; p.(Arg594Cys), retains partial spastin function in the cytoplasm, allowing more effective microtubule depolymerization and lower tubulin levels [37]. These genotype–phenotype correlations indicate that specific mutations differentially impact spastin's ability to localize and function, influencing microtubule integrity, mitochondrial dynamics, and cytoskeletal remodeling [3, 4, 12, 13].

The impact of spastin mutations on tubulin dynamics varies depending on their effects on protein stability, localization, and enzymatic activity [6, 42]. Impaired spastin function can result in either an accumulation of stabilized microtubules, reducing cytoskeletal flexibility and intracellular trafficking, or excessive microtubule degradation, compromising cell integrity and organelle positioning [12, 13, 42]. These disruptions may underlie the defects observed in mitochondrial morphology and adhesion properties among patients [41, 42]. However, further studies are needed to determine whether these effects arise from altered protein folding, defective trafficking, or loss of ATPase activity, ultimately contributing to the variability in disease severity among patients [12, 13, 23].

In conclusion, our study advances the understanding of the phenotypic spectrum related to *SPAST* variants, supporting their inclusion within the cerebral palsy (CP) spectrum due to early‐onset symptoms and an extended phenotype beyond muscular spasticity. This research not only broadens the molecular understanding of complex hereditary spastic paraplegia (HSP) but also expands the known inheritance patterns of *SPAST*‐related disorders. While symptom severity and onset vary in the biallelic form, our preliminary functional studies did not reflect the same extent, highlighting the need for further investigation. Additional cellular mechanisms likely contribute to the disorder's pathogenesis, highlighting the importance of addressing these knowledge gaps in order to gain a comprehensive understanding and to enable the development of potential therapeutic interventions.

## Author Contributions

G.A.N., M.R., Y.J., C.H.‐D., J.M., R.U., A.N., C.O., D.N.‐B., M.S.: composition of original draft and formal analysis. I.G., R.J.R., R.B., A.S., L.A., A.C., E.G.K., L.M., M.L.R.A.: analysis and data collection. G.A.N., M.R., R.U., D.N.‐B., M.S.: conceptualization, writing, review, editing. All authors have read and approved the final version of this manuscript.

## Conflicts of Interest

The authors declare no conflicts of interest.

## Supporting information


**Figure S1:** Multiple alignment of C‐terminal portion of spastin protein in a variety of model organism from human to yeast. Protein code and organism is indicated in each line (with Clustal Omega). Highlighted, mutated residues identified here.

**Figure S2:** Detection of allelic imbalance in *SPAST* mRNA. The maternal allele (c.1780C>T in exon 17) is observed in a hemizygous state, appearing as homozygous, whereas the paternal variant (c.1325A>T in exon 11) is not detectable at the mRNA level.

**Figure S3:** Electropherograms showing a homozygous *SPAST* mutation (c.1370C>T) in Individual C1 and heterozygous carrier status in both parents.

**Table S1:** Variants identified in the individuals described here.

**Appendix S1:** In vivo confocal time‐lapse movies, recorded over approximately 10 min with a 22‐s interval between frames, illustrate the dynamic behavior and morphology of mitochondria and cell membranes in fibroblasts from both healthy controls and patients. Cells were labeled with MitoTracker Green (mitochondria, green), CellMask Deep Red (cell membrane, red), and Hoechst (nuclei, blue). These recordings reveal clear differences in filopodia‐like structures and mitochondrial morphology between control and patient‐derived fibroblasts.

## Data Availability

All pertinent data and methodologies are detailed in the article and Supporting Information. Data may be accessible to researchers for approved purposes upon contacting the corresponding authors.
