# Supplementary material for: Expanding Hereditary Spastic Paraplegias Limits: Biallelic SPAST Variants in Cerebral Palsy Mimics
Source: Ann Clin Transl Neurol. 2025 Sep 26;13(1):108–21. doi: 10.1002/acn3.70206 (PMC12790158; doi:10.1002/acn3.70206)
Supplement: Supplementary file 1 — Figure S1: Multiple alignment of C‐terminal portion of spastin protein in a variety of model organism from human to yeast. Protein code and organism is indicated in each line (with Clustal Omega). Highlighted, mutated residues identified here. [file ACN3-13-108-s002.pdf]

Supplementary Figure 1.

```
                                                    A425             E442             A457
Q9UBP0_SPAST_HUMAN             ATFFNISAASLTSKYVGEGEKLVRALFAVARELQPSIIFIDEVDSLLCERRE--GEHDAS      458
XP_001164367.1_P. troglodytes  ATFFNISAASLTSKYVGEGEKLVRALFAVARELQPSIIFIDEVDSLLCERRE--GEHDAS      458
NP_001075060.1_B. taurus       ATFFNISAASLTSKYVGEGEKLVRALFAVARELQPSIIFIDEVDSLLCERRE--GEHDAS      456
Q719N1.3_S. scrofa             ATFFNISAASLTSKYVGEGEKLVRALFAVARELQPSIIFIDEVDSLLRERRE--GEHDAS      458
XP_023475046.1_E. caballus     ATFFNISAASLTSKYVGEGEKLVRALFAVARELQPSIIFIDEVDSLLCERRE--GEHDAS      426
XP_008762710.1_R. norvegicus   ATFFNISAASLTSKYVGEGEKLVRALFAVARELQPSIIFIDEVDSLLCERRE--GEHDAS      424
XP_031807091.1_S. harrisii     ATFFNISAASLTSKYVGEGEKLVRALFAVARELQPSIIFIDEVDSLLCERRE--GEHDAS      461
NP_001026232.2_G. gallus       ATFFNISAASLTSKYVGEGEKLVRALFAVARELQPSIIFIDEVDSLLCERRE--GEHDAS      455
XP_012818464.1_X.tropicalis    ATFFNISAASLTSKYVGEGEKLVRALFSVARELQPSIIFIDEVDSLLCERRE--GEHDAS      445
NP_998080.2_D. rerio           ATFFNISAATLTSKYVGEGEKLVRALFAVARELQPSIIFIDEIDSLLCERRE--GEHDAS      411
NP_001303437.1_D. melanogaster ATFLNISAASLTSKYVGDGEKLVRALFAVARHMQPSIIFIDEVDSLLSERSS--SEHEAS      330
NP_001323444.1_A. thaliana     ATFFNVSASSLTSKWVGEAEKLVKTLFQVAISRQPSVIFMDEIDSIMSTRST--SENEAS      199
XP_637373.1_D. discoideum      VTFFSISSSSLTSKYVGDGEKLVRALFAVATHFQPSIIFIDEIDSLLTERSS--NESEAS      500
NP_594206.1_S.pombe            SVFFSISASSLTSKFLGESEKLVRALFTLAKKLSPSIIFVDEIDSLLSARSSDGNEHETS      577
                                .*:.:*:::****::*:.****::** :*   .**:**:**:**::  *    .* ::*

Q9UBP0_SPAST_HUMAN             RRLKTEFLIEFDGVQS------AG-DDRVLVMGATNRPQELDEAVLRRFIKRVYVSLPNE      511
XP_001164367.1_P. troglodytes  RRLKTEFLIEFDGVQS------AG-DDRVLVMGATNRPQELDEAVLRRFIKRVYVSLPNE      511
NP_001075060.1_B. taurus       RRLKTEFLIEFDGVQS------AG-DDRVLVMGATNRPQELDEAVLRRFTKRVYVSLPNE      509
Q719N1.3_S. scrofa             RRLKTEFLIEFDGVQS------AG-DDRVLVMGATNRPQELDEAVLRRFIKRVYVSLPNE      511
XP_023475046.1_E. caballus     RRLKTEFLIEFDGVQS------AG-DDRVLVMGATNRPQELDEAVLRRFTKRVYVSLPNE      479
XP_008762710.1_R. norvegicus   RRLKTEFLIEFDGVQS------AG-DDRVLVMGATNRPQELDEAVLRRFIKRVYVSLPNE      477
XP_031807091.1_S. harrisii     RRLKTEFLIEFDGVQS------AG-DDRVLVMGATNRPQELDEAVLRRFIKRVYVSLPNE      514
NP_001026232.2_G. gallus       RRLKTEFLIEFDGVQS------SG-EDRILVMGATNRPQELDDAVLRRFTKRVYVSLPNE      508
XP_012818464.1_X.tropicalis    RRLKTEFLIEFDGVQS------GG-DDRVLVMGATNRPQELDDAVLRRFTKRVYVSLPNE      498
NP_998080.2_D. rerio           RRLKTEFLIEFDGVQS------GG-DERVLVMGATNRPQELDEAVLRRFAKRIYVALPTE      464
NP_001303437.1_D. melanogaster RRLKTEFLVEFDGLPG------NPDGDRIVVLAATNRPQELDEAALRRFTKRVYVSLPDE      384
NP_001323444.1_A. thaliana     RRLKSEFLIQFDGVTS------NP-DDLVIIIGATNKPQELDDAVLRRLVKRIYVPLPDS      252
XP_637373.1_D. discoideum      RRLKTEILVQFDGART------NG-DERVLVMGATNRPEDLDDAALRRLVKRIYVGLPEL      553
NP_594206.1_S.pombe            RRIKTEFLIQWSSLARAAASRQTADHPRVLVLAATNLPWCIDDAARRRFVRRTYIPLPDE      637
                               **:*:*:*:::..               ::::.*** *  :*:*. **: :* *: **
                                                                         K554
Q9UBP0_SPAST_HUMAN             ETRLLLLKNLLCKQGSPLTQKELAQLARMTDGYSGSDLTALAKDAALGPIRELKPEQVKN      571
XP_001164367.1_P. troglodytes  ETRLLLLKNLLCKQGSPLTQKELAQLARMTDGYSGSDLTALAKDAALGPIRELKPEQVKN      571
NP_001075060.1_B. taurus       ETRLLLLKNLLCKQGSPLTQKELAQLARMTNGYSGSDLTALAKDAALGPIRELKPEQVKN      569
Q719N1.3_S. scrofa             ETRLLLLKNLLCKQGSPLTQKELAQLARLTDGYSGSDLTALAKDAALGPIRELKPEQVKN      571
XP_023475046.1_E. caballus     ETRLLLLKNLLGKQGSPLTQKELAQLARMTDGYSGSDLTALAKDAALGPIRELKPEQVKN      539
XP_008762710.1_R. norvegicus   ETRLLLLKNLLCKQGSPLTQKELAQLARMTDGYSGSDLTALAKDAALGPIRELKPEQVKN      537
XP_031807091.1_S. harrisii     ETRLLLLKNLLSKQGSPLTQKELAQLARMTEGYSGSDLTALAKDAALGPIRELKPEQVKN      574
NP_001026232.2_G. gallus       ETRLILLKNLLSKQGSPLTQKELAQLARMTDGYSGSDLTALAKDAALGPIRELKPEQVKN      568
XP_012818464.1_X.tropicalis    ETRLLLLKNLLSKQGNPLNEKELTQLSRLTEGYSGSDITALAKDAALGPIRELKPEQVKN      558
NP_998080.2_D. rerio           ETRLKLLKNLLSKHRNPLSQKELSQLARLTDGYSGSDLTSLAKDAALGPIRELKPEQVRN      524
NP_001303437.1_D. melanogaster QTRELLLNRLLQKQGSPLDTEALRRLAKITDGYSGSDLTALAKDAALEPIRELNVEQVKC      444
NP_001323444.1_A. thaliana     NVRKLLFKTKLKCQPHSLSDGDIDKIVKETEGYSGSDLQALCEEAAMMPIRELGANI-LT      311
XP_637373.1_D. discoideum      ETRLQIIQHLLVGQRHSLTKQQINSLAEVTQGYSGFDLAALCKDAAYEPIRRLGIGI-KD      612
NP_594206.1_S.pombe            TTRRLHLNNLLKYQKHSLSLEDIEAIVKATEYYSGSDLTALAKDAAMGPLRSLGESL-LF      696
                                .*   ::  *  :   *    :  : . *: *** *: :*.::**  *:* *
                                                    R594
Q9UBP0_SPAST_HUMAN             MSASEMRNIRLSDFTESLKKIKRSVSPQTLEAYIRWNKDFGDTTV-      616
XP_001164367.1_P. troglodytes  MSASEMRNIRLSDFTESLKKIKRSVSPQTLEAYIRWNKDFGDTTV-      616
NP_001075060.1_B. taurus       MSASEMRNIRLSDFTESLKKIKRSVSPQTLEAYIRWNKDFGDTTV-      614
Q719N1.3_S. scrofa             MSASEMRNIRLSDFTESLKKIKRSVSPQTLEAYIRWNKDFGDTTV-      616
XP_023475046.1_E. caballus     MSASEMRNIRLSDFTESLKKIKRSVSPQTLEAYIRWNKDFGDTTV-      584
XP_008762710.1_R. norvegicus   MSASEMRNIRLSDFTESLKKIKRSVSPQTLEAYIRWNKDFGDTTV-      582
XP_031807091.1_S. harrisii     MSASEMRNIRLSDFTESLKKIKRSVSPQTLEAYIRWNKDFGDTTV-      619
NP_001026232.2_G. gallus       MSASEMRNIKLSDFTESLKKIKRSLSPQTLEAYIRWNKDFGDTTV-      613
XP_012818464.1_X.tropicalis    MAASEMRNIKYSDFLSSLKKIKCSVSPSTLESYIRWNKEFGDTTV-      603
NP_998080.2_D. rerio           MSAHEMRDIRISDFLESLKRIKRSVSPQTLDQYVRWNREYGDTTGV      570
NP_001303437.1_D. melanogaster LDISAMRAITEQDFHSSLKRIRRSVAPQSLNSYEKWSQDYGDITI-      489
NP_001323444.1_A. thaliana     IQANKVRSLRYDDFRKSMAVIRPSLSKSKWEELERWNSEFGSN---      354
XP_637373.1_D. discoideum      LELNEISLISFKDFANSLKQIRPSVTSQSLKSFEKWNQKFGTI---      655
NP_594206.1_S.pombe            TKMESIRPINLDDFKTSIKVIRPSVNLQGLERYSEWDKEFGSQGH-      741
                                    :  :  .**  *:  *: *:  .  .   .*. .:*
```

Multiple alignment of C-terminal portion of spastin protein in a variety of model organism from human to yeast. Protein code and organism is indicated in each line (with Clustal Omega). Highlighted, mutated residues identified here.
