# Supplementary figures and images for: Expanding Hereditary Spastic Paraplegias Limits: Biallelic SPAST Variants in Cerebral Palsy Mimics
Source: Ann Clin Transl Neurol. 2025 Sep 26;13(1):108–21. doi: 10.1002/acn3.70206 (PMC12790158; doi:10.1002/acn3.70206)

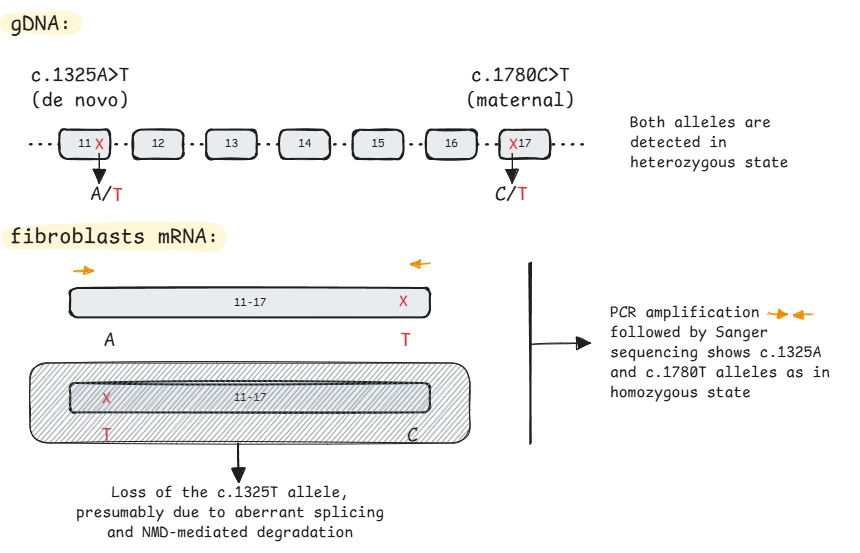

Supplement: Supplementary file 2 — Figure S2: Detection of allelic imbalance in SPAST mRNA. The maternal allele (c.1780C>T in exon 17) is observed in a hemizygous state, appearing as homozygous, whereas the paternal variant (c.1325A>T in exon 11) is not detectable at the mRNA level. [file ACN3-13-108-s001.png]

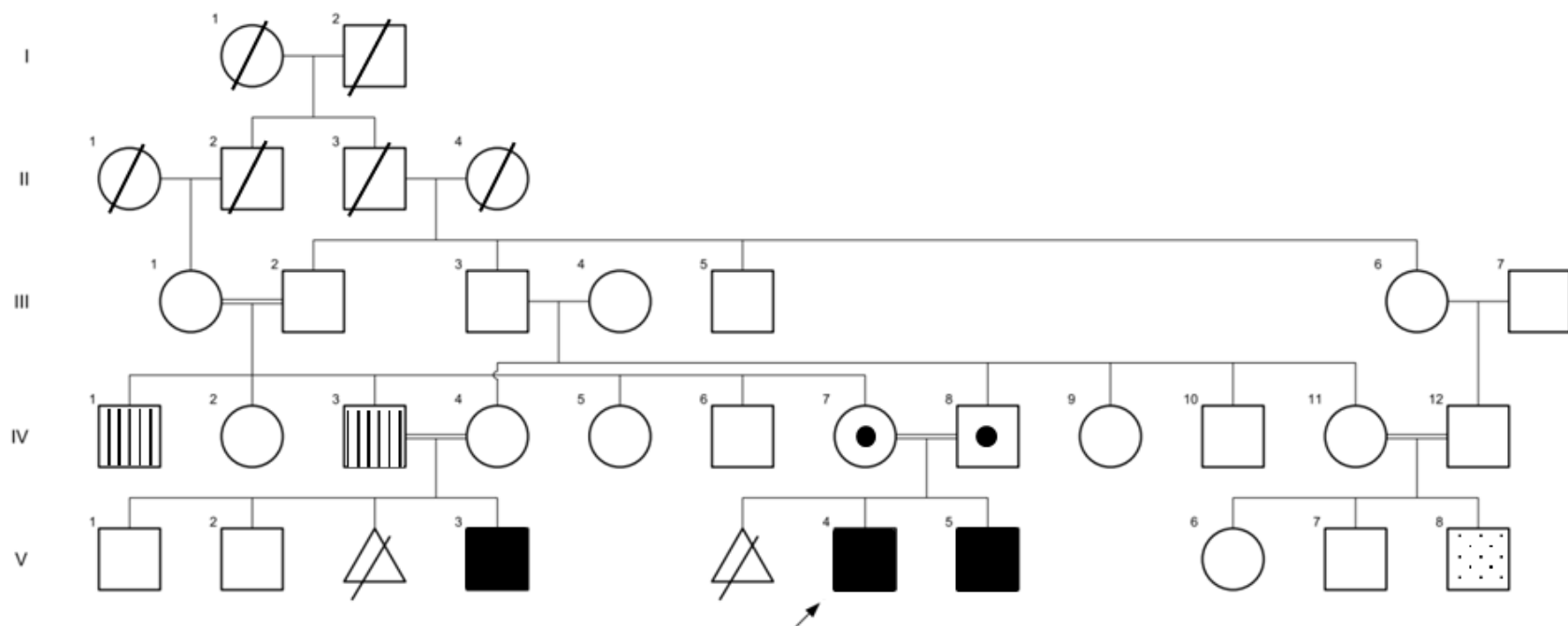

V 4

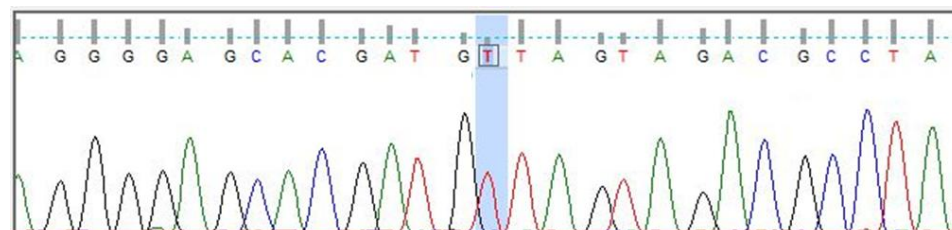

IV 7

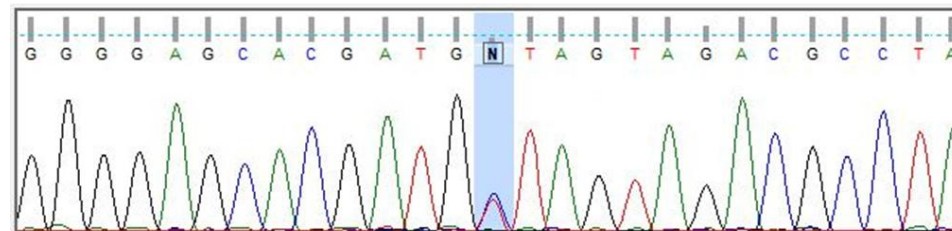

IV 8

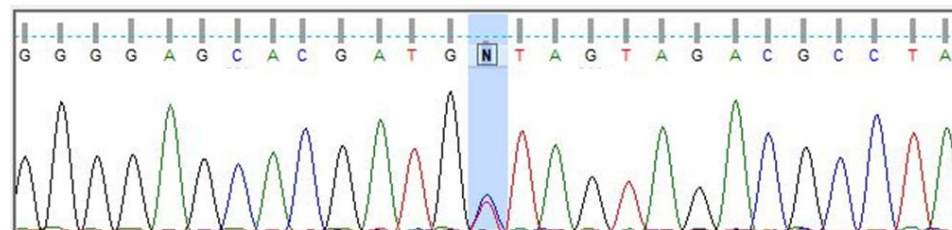

Supplement: Supplementary file 3 — Figure S3: Electropherograms showing a homozygous SPAST mutation (c.1370C>T) in Individual C1 and heterozygous carrier status in both parents. [file ACN3-13-108-s003.pdf]
