# Supplementary material for: Expanding Hereditary Spastic Paraplegias Limits: Biallelic SPAST Variants in Cerebral Palsy Mimics
Source: Ann Clin Transl Neurol. 2025 Sep 26;13(1):108–21. doi: 10.1002/acn3.70206 (PMC12790158; doi:10.1002/acn3.70206)
Supplement: Supplementary file 4 — Table S1: Variants identified in the individuals described here. [file ACN3-13-108-s004.pdf]

| Case | Zigosity | gDNA[(1)] | cDNA | protein | CADD | DANN | SIFT (Score) | PolyPhen2 Score | ClinVar (ID) | GnomAD [(2)] | Other variants in same residue [(3)] | PMID | ACMD Classification |
|---|---|---|---|---|---|---|---|---|---|---|---|---|---|
| A1 | Homozygous (paternal/maternal) | 2:32144980 | c.1660A>G | p.(Lys554Glu) | 23.6 | 0.9983 | D (0.002) | PD (0.613) | Absent | Absent | Lys554_Ala556delinsThr | 30476002 | LP (PS3, PM1, PM2, PP3) |
| B1 | Heterozygous (maternal) | 2:32136880 | c.1325A>T | p.(Glu442Val) | 32 | 0.9935 | D(0) | PD (1) | LP (945131) | Absent | Glu442Ala; Asp; Lys; STOP | 16832076; 31157359; 17957230; 16240363; 25525159 | LP (PM1, PM2, PM5, PP3, PP5) |
| | Heterozygous (*de novo* ) | 2:32154425 | c.1780C>T | p.(Arg594Cys) | 23.8 | 0.9954 | B (0.054) | PD(0.906) | VUS (1417996) | 0.0000075 | Arg594fs | 34008892; 26671083 | LP (PS3, PM1, PM2, PP3) |
| C1 | Heteroz | 2:32136591 | c.1274C>T | p.(Ala425Val) | 29 | 0.9991 | D (0.001) | PD (1) | Absent | Absent | Ala425Pro; fs | 15841487; 30489674; 31594988; 34782662 | LP (PM1, PM2, PM5, PP3) |
| | Heteroz | 2:32136925 | c.1370C>T | p.(Ala457Val) | 31 | 0.9991 | D (0.003) | PD (1) | Absent | Absent | | 25577683 | LP (PM1, PM2, PP3, PP5) |

(1) References to hg38; (2) gromAD 4.0 Accessed April 2025 (3) According to HGMD® Professional 2025.1 accesed April 2025
